# Navigating the Diagnosis and Management of Rheumatoid Arthritis in the Context of Sickle Cell Disease: A Dual Challenge

**DOI:** 10.7759/cureus.44615

**Published:** 2023-09-03

**Authors:** Akhil Sugandhi, Kreetee Dubey, Suman Panda, Zaid Nafe

**Affiliations:** 1 General Medicine, Tata Main Hospital, Jamshedpur, IND; 2 Gastroenterology, Tata Main Hospital, Jamshedpur, IND

**Keywords:** anemia, pain crisis, vascular-occlusive crisis, rheumatoid arthritis (ra), sickle cell disease (scd)

## Abstract

Sickle cell disease is a common condition in the eastern part of India and can often present with pain crisis, vasculo-occlusive crisis and anemia. These patients seldom have coexisting rheumatological illnesses like rheumatoid arthritis that are camouflaged with the pain crisis of sickle cell disease, leading to a delay in the diagnosis and a delay in initiating the treatment which leads to a poorer quality of life. Herein we discuss a case of sickle cell disease presenting concomitantly with the features of rheumatoid arthritis and the challenges faced in the diagnosis and treatment.

## Introduction

Sickle cell disease is caused by a point mutation in the beta-globin gene leading to the replacement of glutamic acid to valine in the sixth position of the beta chain of hemoglobin. Rheumatoid arthritis is an autoimmune chronic inflammatory disease that often presents with early morning stiffness and symmetrical small joint pains. Patients with rheumatoid arthritis may have cartilage and bone destruction that leads to permanent disability and reduced quality of life. The joint pains in rheumatoid arthritis are sometimes masked by the pain crisis of sickle cell disease which leads to a diagnostic dilemma. Besides, there is a contrast in the treatment of rheumatoid arthritis and sickle cell disease that also leads to a treatment dilemma [1].

## Case presentation

**Figure 1:**
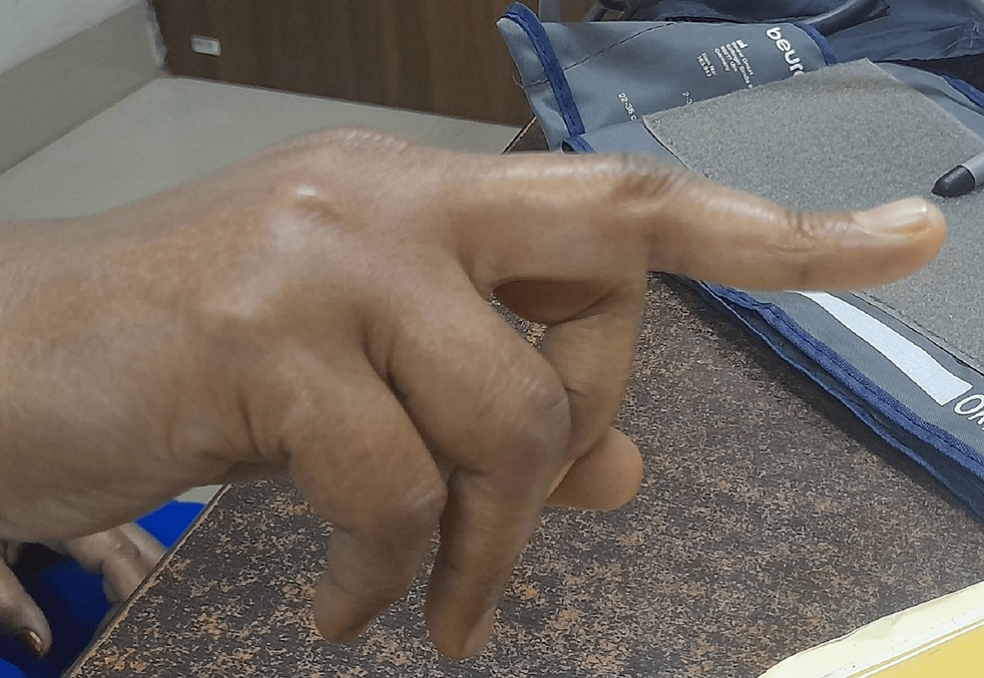
Boutonniere deformity of the middle finger of the right hand.

A 36-year-old female patient presented to us with multiple joint pains and weakness for a duration of seven days. The patient had a confirmed history of sickle cell disease spanning over the course of 11 years. On this occasion, the patient presented with complaints similar to those experienced in the past. Previous episodes had been managed by administering analgesics to alleviate the pain crisis. Upon further inquiry into her symptoms, she disclosed a medical history involving stiffness during the early morning hours and symmetrically distributed joint pain affecting the small joints of her hands. On general examination, her vitals were stable. She had tender and swollen proximal interphalangeal joints and metacarpophalangeal joints in both hands. She also had boutonniere deformity which was quite evident on examination (Figure 1). There was no associated rash or edema. There was no family history of similar complaints. 

Her laboratory investigations revealed a positive rheumatoid factor and increased levels of erythrocyte sedimentation rate (ESR) and C-reactive protein (CRP). Her anti-cyclic citrullinated peptide, antinuclear antibodies and human leukocyte antigen B27 (HLA-B27) were negative. Her hemoglobin levels were 6.4 gm% which was microcytic hypochromic type. Her Disease Activity Score (DAS-28) was 5.1. She was transfused three units of packed red blood cells (RBC). She was treated with a short course of non-steroidal anti-inflammatory drugs (NSAIDs), methotrexate and hydroxychloroquine sulphate and showed improvement in her pains in about seven days and was discharged. She is being followed up regularly to monitor her blood picture and to titrate the dose of disease-modifying antirheumatic drugs (DMARDs).

## Discussion

The diagnosis of rheumatoid arthritis in a patient with sickle cell disease poses a challenge due to the similarities in their presentation. Rheumatoid arthritis is a chronic inflammatory disease that is characterised by cartilage damage, synovitis and juxta-articular bone destruction [2]. Hemoglobin S in sickle cell disease causes the polymerization of hemoglobin in low oxygen conditions which in turn leads to dehydration of RBC which causes sickling [3]. Sickled RBC can lead to blockage of the blood flow which may lead to vasulo-occlusive and pain crisis [4]. Rheumatoid arthritis and sickle cell disease tend to share a commonality of the raised inflammatory cytokines. The inflammatory markers released during the vasculo-occlusive crisis may lead to early development of rheumatoid arthritis. However the recurrent occurrence of pain crisis may hinder the early clinical detection of rheumatoid arthritis which leads the clinician to a diagnostic dilemma.

Once the diagnostic dilemma is overcome, the next barrier a clinician faces is the treatment dilemma. The use of steroids in rheumatoid arthritis is for transient pain relief before the action of DMARDs sets in. However, steroids if used in rheumatoid arthritis with sickle cell disease can lead to vasculo-occlusive crisis and bone infarcts. Hydroxyurea can reduce the white blood cell and reticulocyte counts and can lead to opportunistic infections [5]. The use of hydroxyurea with steroids remains a topic of debate as it can further lead to immunosuppression. NSAIDs tend to worsen the renal damage that may be caused by sickle cell disease and this complicates the treatment for pain [6]. There is no known adverse effect of concomitant use of hydroxyurea and hydroxychloroquine [7]. A study conducted by Brandalise et al. showed that there was little impact of methotrexate on vasculo-occlusive crises in patients with sickle cell disease who were on hydroxyurea. However, the pain due to avascular necrosis is reduced [8]. It is essential to monitor the complete blood picture while using methotrexate with hydroxyurea as it can cause significant thrombocytopenia. Regular monitoring of the liver function and renal function may be essential. Concomitant use of sulfasalazine and hydroxyurea has not been reported [9].

## Conclusions

The musculoskeletal features of sickle cell disease can mask the clinical features of rheumatoid arthritis, leading to diagnostic challenges and once diagnosed can present with challenges in the treatment. Steroids if used in rheumatoid arthritis with sickle cell disease should be used with caution. Regular monitoring of the hemoglobin, platelets, liver and renal function is required with using DMARDs in rheumatoid arthritis and sickle cell disease. The diagnosis of rheumatoid arthritis is rarely considered and overlooked because of the musculoskeletal symptoms of sickle cell crises. Underlying inflammatory mechanisms of both sickle cell disease and rheumatoid arthritis may mask the clinical manifestation of each. Prompt use of DMARDs improves the quality of life and prevents further damage. Long-term research to study the quality of life and clinical course is needed in detection of rheumatoid arthritis in sickle cell disease.
